# Plexiform Neurofibroma: A Case Report

**DOI:** 10.7759/cureus.65747

**Published:** 2024-07-30

**Authors:** Kshitij Bang, Ramakrishna Shenoi, Alvina V Waghchoure

**Affiliations:** 1 Oral and Maxillofacial Surgery, VSPM (Vidya Shikshan Prasarak Mandal) Dental College and Research Centre, Nagpur, IND

**Keywords:** nf1, von recklinghausen disease, neurofibroma, plexiform neurofibroma, neurofibromatosis type 1

## Abstract

Neurofibromatosis is a group of genetic disorders that primarily impact the growth of neural tissues, leading to multiple tumors on nerve tissues in the brain, spinal cord, and peripheral nerves. As an autosomal dominant condition, it involves mutations in the neurofibromatosis type 1 (NF1) tumor-suppressor gene, inherited in a recessive manner. Plexiform neurofibroma is a rare manifestation. It is a benign peripheral nerve sheath tumor that grows beneath the skin or deeper within tissues without clear boundaries. The diverse presentations of NF1 necessitate careful, personalized medical management to address the disorder's effects on various organs. Due to its progressive nature, early diagnosis is crucial to prevent complications. Comprehensive care, including psychological support and long-term monitoring, is essential for enhancing the quality of life of NF1 patients. By adopting a proactive and holistic approach, healthcare providers can better assist patients in managing this complex condition.

## Introduction

Neurofibromatosis is the term used to describe a group of genetic disorders that primarily affect the cell growth of neural tissues [1]. It is an autosomal dominant disorder that results in the development of numerous tumors on nerve tissues, such as those in the brain, spinal cord, and peripheral nerves. Three types of neurofibromatosis have been recognized, namely, neurofibromatosis type 1 (NF1), neurofibromatosis type 2 (NF2), and schwannomatosis. The most common form, i.e., NF1, makes up 96% of all cases and is marked by the presence of neurofibromas (tumors on peripheral nerves), which can cause skin abnormalities and bone deformities [2].

Plexiform neurofibroma is a rare tumor that only occurs in 5-15% of individuals with NF1 [1,3]. It is a peripheral nerve sheath tumor that is benign in origin and not well-defined and proliferates beneath the skin or deeper within the tissues [1]. The NF1 gene, associated with the disease, is found on chromosome 17 at the 17q11.2 locus and encodes the protein neurofibromin. This gene acts as a tumor suppressor gene by downregulating the RAS gene product. When mutated, it leads to the growth of multiple neurofibromas and other tumors [3]. These tumors are painless, grow slowly, and infiltrate locally. Their consistency is often described as being similar to a "bag of worms" [1].

In our case report, we describe our surgical experience in managing a case of plexiform neurofibroma involving the midface, causing soft tissue deformity and gross deformation of the face, specifically the nose and lip, leading to facial asymmetry.

## Case presentation

A 10-year-old boy visited the outpatient department of oral and maxillofacial surgery at Vidya Shikshan Prasarak Mandal’s (VSPM) Dental College and Research Centre, Nagpur with the chief complaint of swelling over the left side of the face for the past six years. The swelling led to gross facial asymmetry. It extended anteroposteriorly from the tip of the nose and the left corner of the mouth to the left angle of the mandible. Superiorly, the swelling was noted 1 cm above the ala tragus line, which extended 1 cm above the inferior border of the mandible. The swelling was not well circumscribed and presented with irregular shapes and ill-defined borders. The dimensions of the swelling noted were approximately 6 x 4 cm.

**Figure 1:**
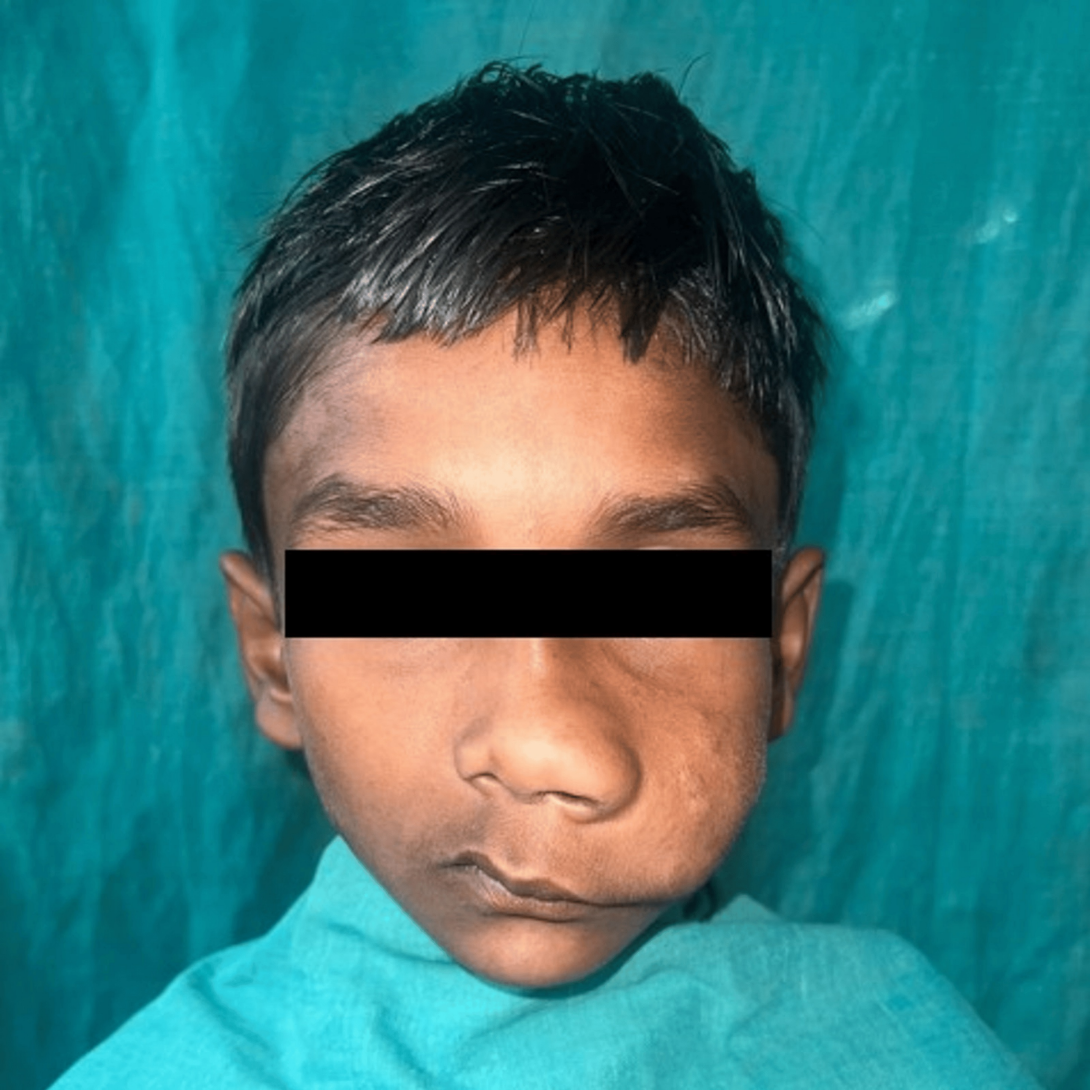
Preoperative image of the case showing plexiform neurofibroma involving the midface.

The swelling increased gradually to attain the noted size and it was progressive in nature. The skin over the swelling was sagging and no tension was noted over the skin. Obliteration of the nasolabial fold was noted over the left side of the face. The swelling primarily involved the lower half of the nose with a bulbous tip and alar flaring accompanied by deviation of the nose toward the right side. Primary involvement of the upper lip resulted in excess tissue in the transverse dimension with caudal displacement of the oral commissure (Figure 1).

**Figure 2:**
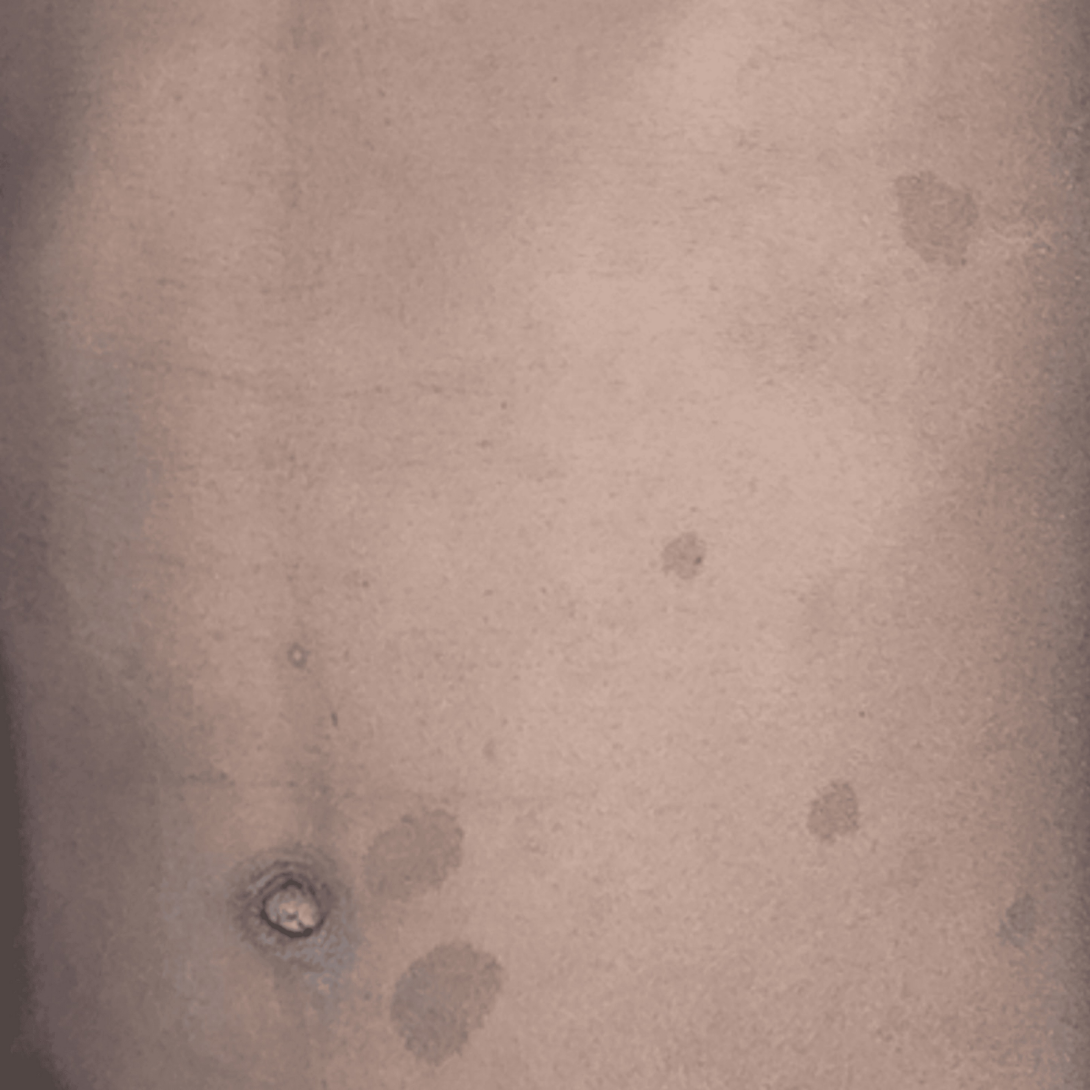
Café-au-lait spots noted over the trunk and the back.

On palpation, no tenderness was associated with swelling. Paresthesia was noted with respect to the left infraorbital nerve. The consistency of the swelling was soft and no fixity of the swelling was noted with the underlying tissues. Intraorally, the swelling extended to the hard palate causing expansion of the palate along with macules noted over the left attached gingiva and the left cheek. Multiple café-au-lait spots were noted over the trunk and the back along with axillary freckling (Figure 2).

**Figure 3:**
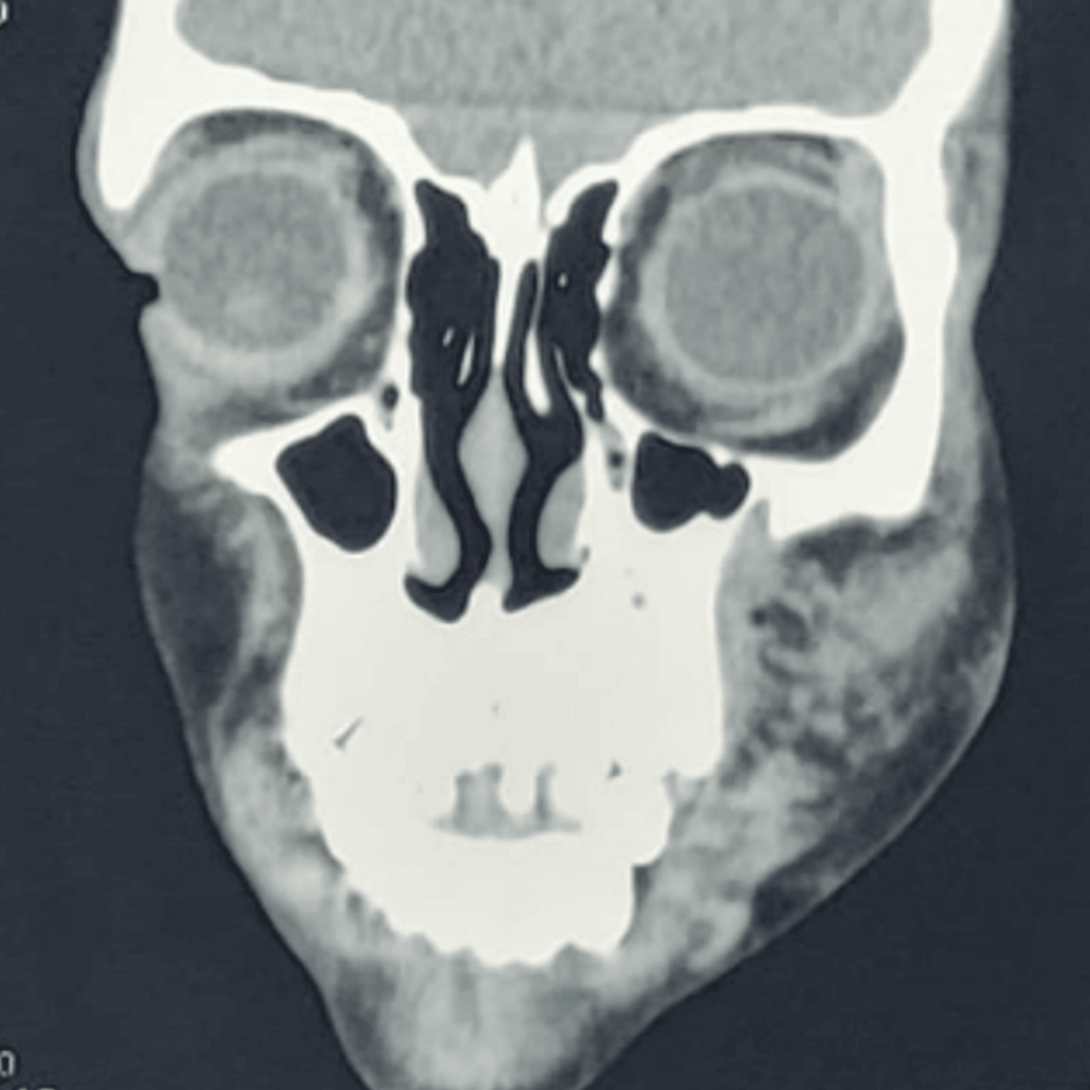
Contrast-enhanced computed tomography image of the lesion.

Contrast-enhanced computed tomography (CECT) was suggestive of a subcutaneous soft tissue density mass lesion measuring 2.6 x 5.1 x 4.8 cm involving the anterior half of the left cheek, nasal ala, nasolabial fold, and the upper lip, which extended inwards up to the anterior aspect of floor of the nose on the left side. Elevation of the overlying skin was noted and the lesion showed post-contrast enhancement. MRI report was suggestive of an altered signal intensity lesion in the left maxillofacial region involving the subcutaneous plane with elevation of overlying skin, and the largest size measuring 3.7 x 5.0 x 4.9 cm (anteroposterior (AP), transverse, and craniocaudal (CC) dimensions, respectively). The lesion appeared isointense on T1 and hyperintense on T2. It extended superiorly up to the orbicularis oculi muscle with maintained fat planes and laterally up to the skin surface causing its elevation. Medially, the lesion involved the nasal ala, nasolabial fold, and upper lip and extended inwards up to the anterior aspect of the nose. The lesion inferiorly extended subcutaneously up to the level of the mandible. Fat planes with orbicularis oris and zygomaticus appeared effaced (Figure 3).

**Figure 4:**
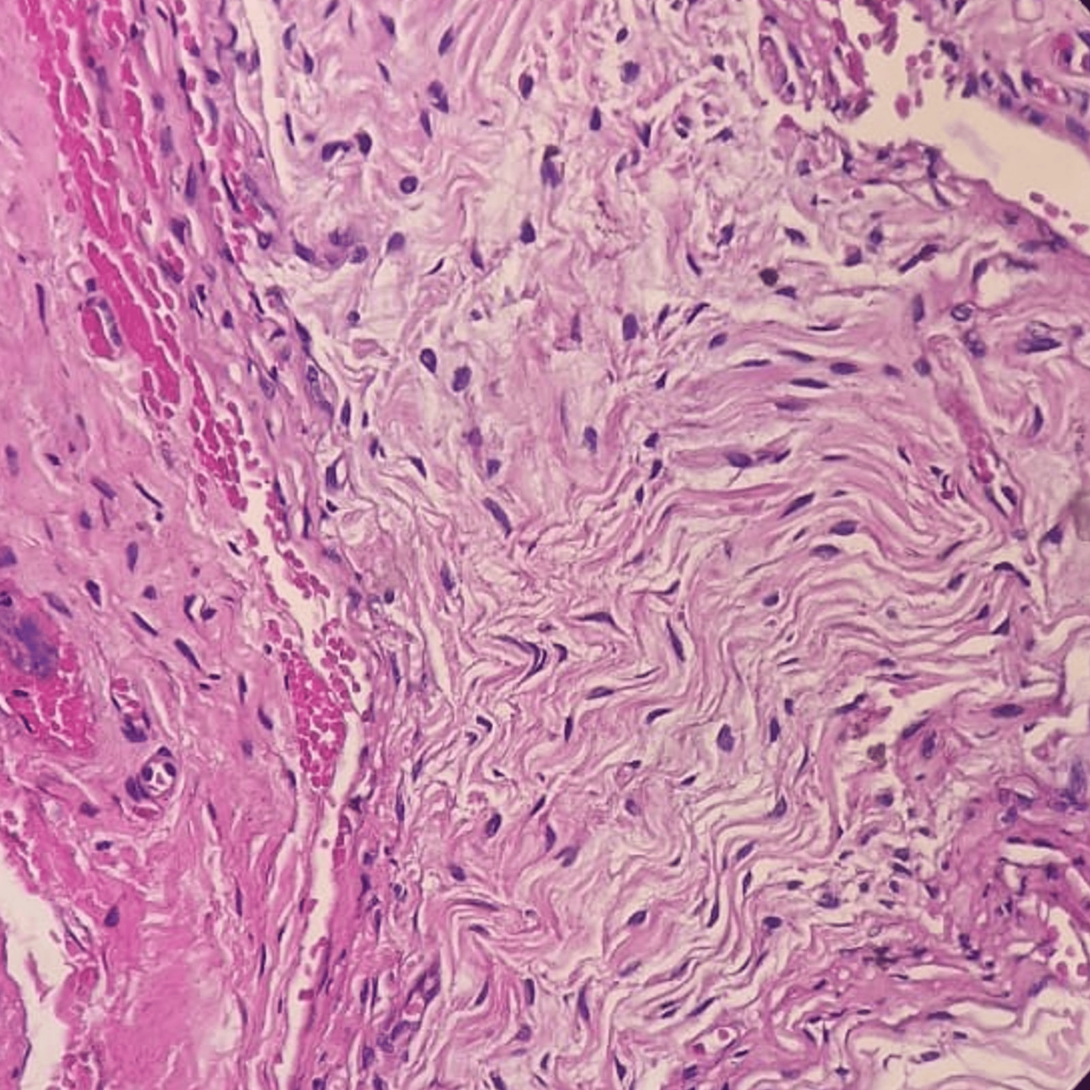
Biopsy image of the lesion. The image shown is in 40x magnification and stained with hematoxylin & eosin (H&E).

The biopsy of the soft tissue suggested keratinized squamous epithelium with atrophy of stratification, and subepithelial connective tissue showed a dense aggregate of neural and plump-shaped fibrotic nests intervened against abundant collagenous background. The features were suggestive of plexiform neurofibroma. The bone biopsy was suggestive of bony trabeculae with intramedullary spindle cell proliferation with elongated nuclei, small prominent nucleoli, and foci of calcification (Figure 4).

There was no history of similar complaints in any family members or distant relatives. Based on the diagnostic criteria mentioned by the National Institutes of Health (Bethesda, USA) and the aforementioned investigations, a conclusive diagnosis of NF1 was made (Table 1).

**Table 1:** Diagnostic criteria for neurofibromatosis type 1 (NF1). * If only café-au-lait macules and freckling are present, the diagnosis is most likely NF1 but exceptionally the person might have another diagnosis such as Legius syndrome. At least one of the two pigmentary findings (café-au-lait macules or freckling) should be bilateral. ** Sphenoid wing dysplasia is not a separate criterion in the case of an ipsilateral orbital plexiform neurofibroma [2].

A: The diagnostic criteria for NF1 are met in an individual who does not have a parent diagnosed with NF1 if two or more of the following are present:
- At least six café-au-lait macules (>5 mm diameter in prepubertal individuals and >15 mm in postpubertal individuals)
- Freckling in axillary or inguinal regions*
- Optic glioma
- At least two Lisch nodules identified by slit-lamp examination or two or more choroidal abnormalities—defined as bright, patchy nodules imaged by optical coherence tomography/near-infrared reflectance imaging
- At least two neurofibromas of any type, or one plexiform neurofibroma
- A distinctive osseous lesion such as sphenoid dysplasia**, anterolateral bowing of the tibia, or pseudarthrosis of a long bone
- A heterozygous pathogenic NF1 variant with a variant allele fraction of 50% in apparently normal tissue such as white blood cells
B: A child of a parent who meets the diagnostic criteria specified in A merits a diagnosis of NF1 if one or more of the criteria in A are present

**Figure 5:**
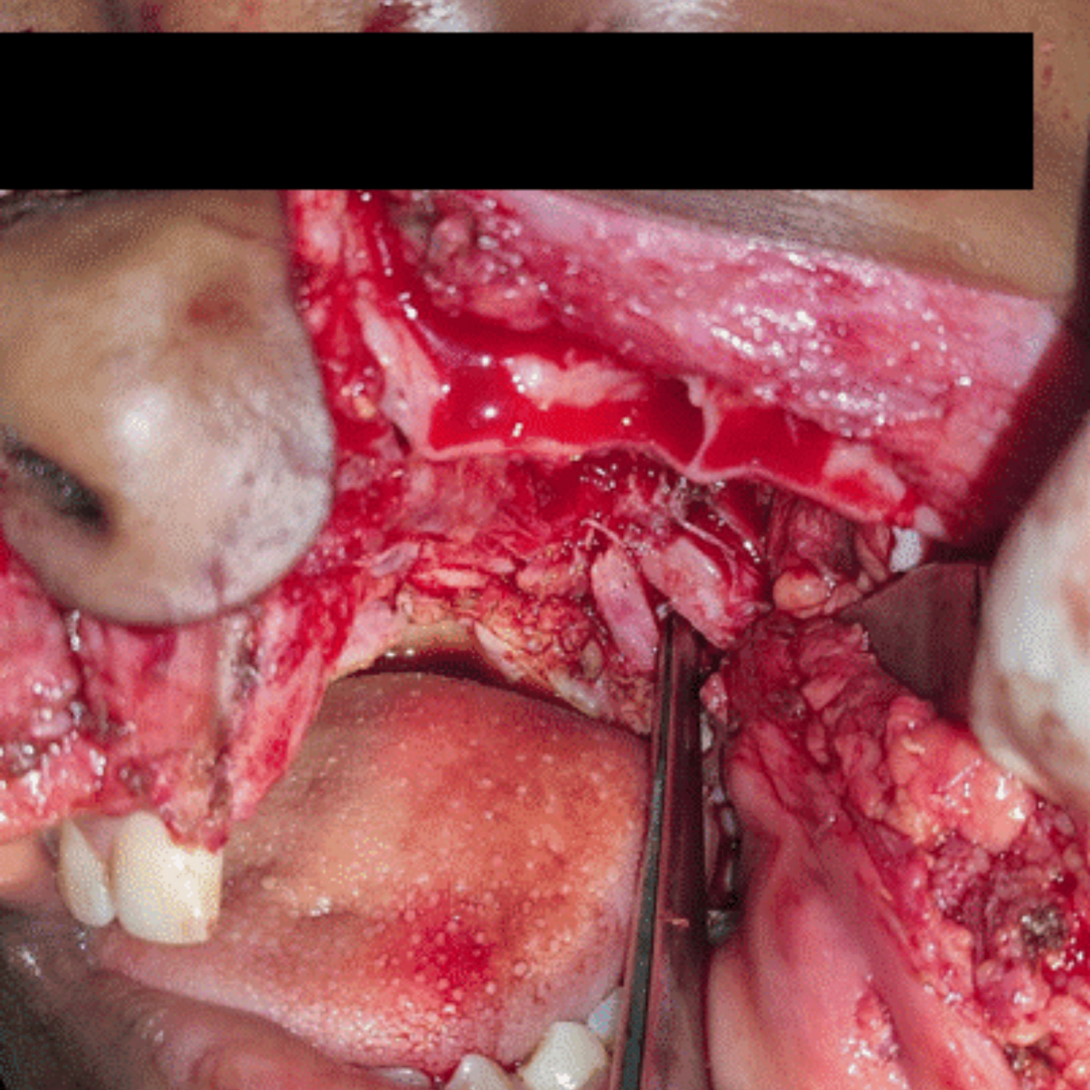
Intraoperative image of the site of the lesion after maxillectomy procedure.

The excision of the tumor was planned under general anesthesia. The Weber Ferguson’s incision was chosen for resection of the tumor. Neurofibromas are not capsulated and have a significant fragile blood supply, which leads to tedious dissection, and difficulty in developing a surgical plane and thus carries a high risk of considerable blood loss. To keep the skin intact, surgical plane and dissection were done carefully in the subcutaneous plane. For tumescent, normal saline was injected in the subcutaneous plane to reduce the blood loss during dissection. Bony margins were exposed and the maxillectomy procedure was carried out (Figure 5).

**Figure 6:**
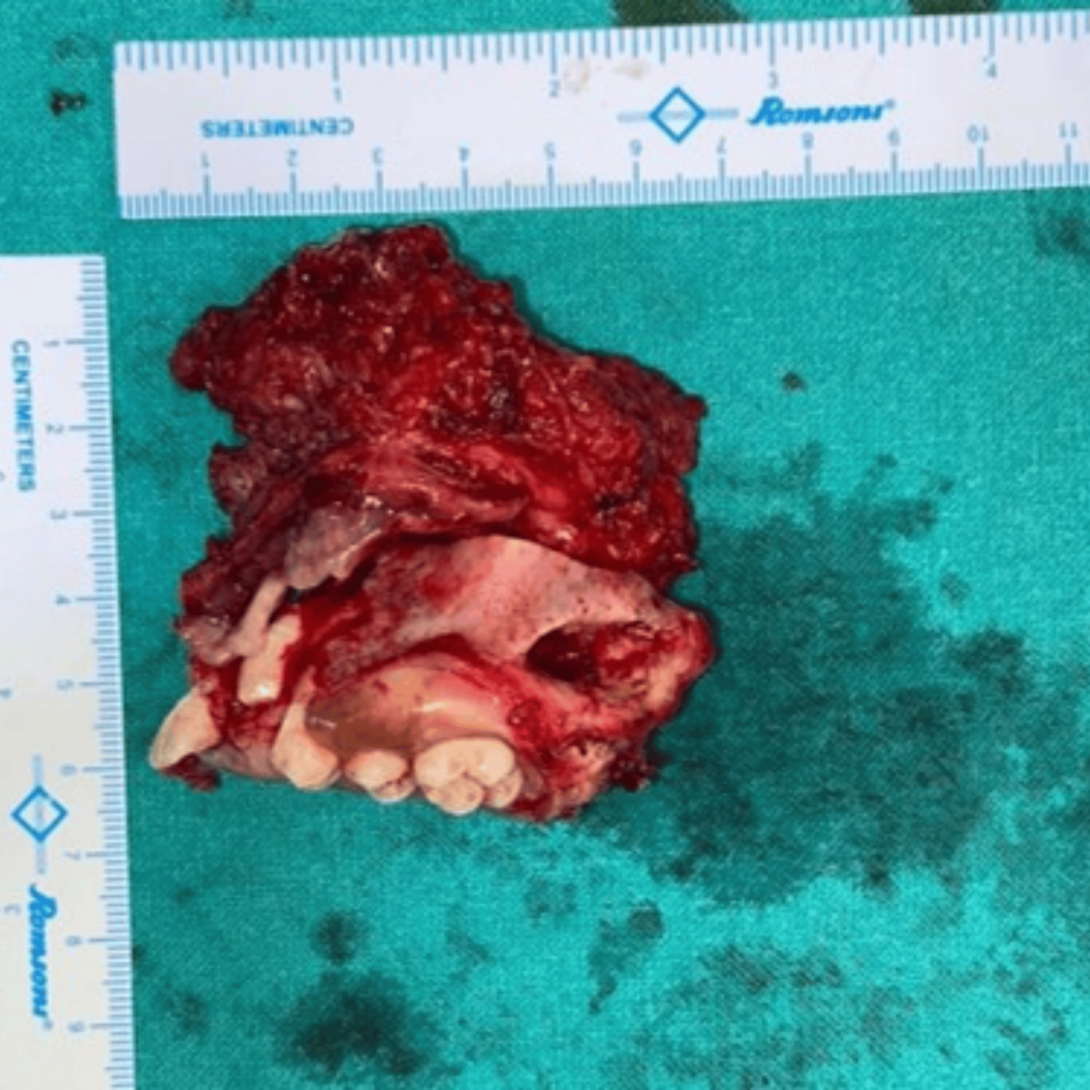
Image of the resected specimen of the lesion.

Considering the growing age of the child and the chances of recurrence of the tumor, no definitive bony reconstruction was done and an obturator prosthesis was fabricated to close the defect created by surgical resection. The resected specimen was sent for further histopathological examination, which re-confirmed the diagnosis (Figure 6).

**Figure 7:**
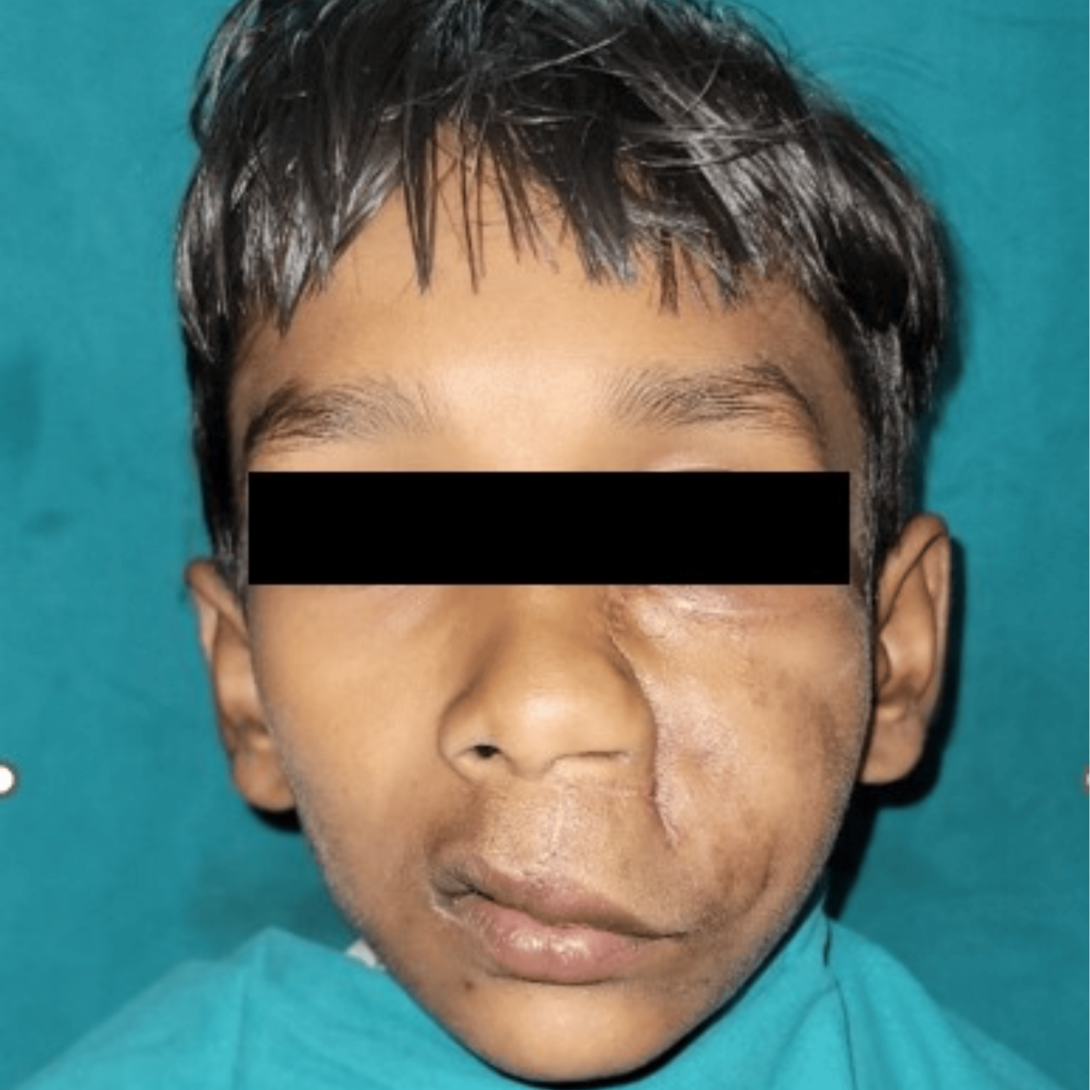
One-month postoperative image.

 The patient was kept on regular follow-up and showed satisfactory healing (Figure 7).

## Discussion

NF1 is a tumor-suppressor gene, with mutations in this gene inherited in a recessive transmission. For the deregulation to occur, both alleles of the gene must be functionally inactivated [4]. Clinically, NF1 may present with café-au-lait spots, axillary freckling, at least two Lisch nodules identified by slit-lamp examination, optic glioma, plexiform neurofibroma, etc., as mentioned in Table 1. Our case presented with café-au-lait spots on the chest and back, axillary freckling, and plexiform neurofibroma on the face, which met the diagnostic criteria for NF1. Diagnosing NF1 in adults is generally straightforward through physical examination. However, in children, café-au-lait macules might be the only noticeable sign for an extended period, often being the most prominent manifestation without typically progressing to malignant tumors [3].

The midface, particularly the nose and upper lip, is frequently impacted in patients with craniofacial neurofibromatosis, significantly affecting their overall aesthetic appearance. Previous discussions on the treatment of the midface in craniofacial neurofibromatosis have been extensively debated in the literature. However, specific attention has been given to the nasolabial region and oral commissures, referred to as "areas of drift," highlighting their critical importance. The neurofibromas affect the midface by two mechanisms: (1) primary cases wherein the midface is affected by direct infiltration of the tumor; (2) secondary cases wherein there is no primary tumor in the midface, but downward traction caused due to neurofibromas in adjacent areas cause significant deformation of the midface, such as the cheek [5].

Plexiform neurofibroma are peculiarly benign in nature but they can be associated with disfigurement, pain, and functional changes. More importantly, they have the potential to become malignant, making it certainly arduous to accurately predict disease progression in an individual. The one consistent feature in all cases of NF1 is its progressive nature, indicating a general trend of disease worsening over time. Surgery is the primary treatment for solitary neurofibromas; however, it is not curative for plexiform neurofibromas ascribed to their invasive nature and location over the midface, which makes the complete resection of the tumor tedious [1].

Plexiform neurofibromas are typically intertwined with normal tissues, posing challenges for surgeons. Additionally, neurofibromas lack a capsule and have a substantial, fragile blood supply, making dissection challenging, carrying a high risk of significant blood loss and a tendency to recur after removal. Surgery is usually only performed if the tumor becomes symptomatic or causes significant disfigurement, particularly in children [6,7]. The literature mentions about two approaches as treatment modalities for the management of neurofibroma, which include either conservative excision or radical excision [1,6]. Conservative excision carries a risk of recurrence whereas radical excision may require extensive reconstruction. The lesion on the midface of our patient caused major aesthetic concern and extended to the palatal region. Hence, the maximum possible excision of the tumor was done by soft tissue excision preserving only the skin flap and a maxillectomy procedure was carried out [8].

The one prominent feature in all cases of NF1 is its progressive nature, leading to a general trend of worsening disease. Therefore, the role of an informed clinician is to diagnose the condition early and inform the patient about potential future complications. Long-term follow-up is essential for these cases. Only a few cases have been documented globally and these procedures need to be carried out in well-equipped facilities with a multidisciplinary team because of the high risks associated with the tumor [9,10]. Psychological counseling and fostering self-confidence in patients can potentially alleviate their suffering and enhance their quality of life. In the end, management should prioritize proactive guidance, genetic counseling, and symptomatic treatment.

## Conclusions

The diverse manifestations of NF1 require vigilant, individualized medical care to effectively manage the disorder's impact on various organs. Given its progressive nature, early diagnosis is paramount to mitigate potential complications. Comprehensive care, including psychological counseling and continuous, long-term follow-up, plays a crucial role in improving the overall quality of life of patients with NF1. By adopting a proactive and holistic approach, healthcare providers can better support patients in navigating the challenges associated with this complex condition.
